# Application of pectinases for recovery of grape seeds phenolics

**DOI:** 10.1007/s13205-016-0537-0

**Published:** 2016-10-17

**Authors:** Petra Štambuk, Dora Tomašković, Ivana Tomaz, Luna Maslov, Domagoj Stupić, Jasminka Karoglan Kontić

**Affiliations:** Department of Viticulture and Enology, Faculty of Agriculture, University of Zagreb, Svetošimunska 25, Zagreb, Croatia

**Keywords:** Enzyme-assisted extraction, Grape marc seeds, Polyphenol compounds, Pectinases, ‘Regent’

## Abstract

Grape marc seeds contain large amounts of different polyphenolic compounds so they can be used for the recovery of these classes of compounds. A new green extraction method for the recovery of phenols from the grape seeds was developed. To provide a high quality extract sourced from natural product by using enzymes as alternative solvents and spending less energy, it is impeccable to call this method “green”. Furthermore, this method was optimized by finding out which conditions provide the best possible results and achieving the maximum recovery of polyphenols from grape seeds. The optimization of the enzyme-assisted extraction of phenols was conducted using the commercially available oenological enzyme preparations with respect to the enzyme dosage, temperature, extraction time, pH value and enzyme preparation by applying the response surface methodology. Optimal conditions were determined using the enzyme preparation Lallzyme EX-V, at the extraction temperature of 48 °C, extraction time of 2 h and 43 min, pH 3.5 and enzyme dosage of 20.00 mg g^−1^. The new optimized extraction method is less expensive, simple, fast, precise and selective for the recovery of simple phenols (monomeric and dimeric form) and since it is based on the environmentally friendly extraction solvent it may provide a valuable alternative to the conventional extraction methods. The obtained extracts can be used for the application in pharmaceutical, food and cosmetic industry.

## Introduction

Grape is one of the most important horticultural crops in the world with an annual production greater than 77 million tons in year 2013, whereof the European countries contribute about 38 %. After vinification, there remains over 3 million tons of grape marc, which is sometimes used as material for obtaining certain biocomponents (FAOSTAT 2013). Grape marc is mainly composed of grape skins (78 %) and grape seeds (16 %) (Dwyer et al. 2014) and contains numerous high-value compounds such as organic acids (tartaric, malic and citric acids), polyphenolic compounds (anthocyanins, flavan-3-ols, and flavonols), aroma compounds, grape seed oil and dietary fibers (Yu and Ahmedna 2013). Among the polyphenolic compounds, grape seeds contain a high amount of monomeric, oligomeric and polymeric forms of flavan-3-ols and hydroxybenzoic acids. Most abundant monomeric flavan-3-ols are epigallocatechin, gallocatechin, catechin, epicatechin and epicatechin-gallate. Polymeric forms or condense tannins are polymers formed by the condensation of monomeric flavan-3-ols. Their properties are defined by the nature of interflavan bonds and monomeric units. In comparison to monomeric and dimeric forms, these compounds do not dissolve in aqueous phases.

The literature showed that, in vitro and/or in vivo, phenols are able to: reduce inflammation, stop the development of tumors, present proapoptotic and anti-angiogenic actions. These compounds can modulate the immune system and prevent osseous disturbance, as well. There is evidence that phenols are able to aid incriminated in osteoporosis, increase the capillary resistance by acting on the constituents of blood vessels, protect the cardiovascular system as well as protect the retina. These compounds are used in numerous sectors of the food industry as natural additives as well as in the cosmetic and pharmaceutical industry (Zillich et al. 2015).

In the production of the food and pharmaceutical products, raw organically obtained materials with a very low content of agrochemical residues are preferred. This is especially highlighted in the viticulture. Due to the high sensitivity of grapevine (*Vitis vinifera* L.) to fungal diseases, this branch of agriculture applies a very high amount of fungicides (EU 2003). To reduce the fungicide application, the trend today is to allow the cultivation of disease resistant grape cultivars obtained by crossing the American species with *V. Vinifera* ones (Reisch et al. 2012). The cultivar ‘Regent’ is one of the newly successful breeds, characterized by a high content of individual polyphenols and especially anthocyanins, flavonols and flavan-3-ols (Karoglan Kontić et al. 2016).

Sample preparation is the crucial process for the polyphenol analysis of grapes. Different conventional solvent extraction techniques have been applied in polyphenol analysis. These techniques are generally based on the use of different toxic and environmentally harmful organic solvents. The extraction methods based on the extraction solvents harmful to human health are scare which restricts the application of the grape seed extracts in pharmaceutical, food and cosmetic industry. Enzyme-assisted extraction (EAE) is a green extraction technique. Compared to the conventional solvent extractions, EAE does not use toxic organic solvents. During the extraction of the grape berry seeds, a degradation of cell walls is mandatory. This process enables the release of cell components to the extraction solvent. The cell wall could be disrupted by the action of different enzymes such as pectinases [pectin methylesterase (PME), pectin lyase (PL) and polygalacturonase (PG)], tannases, cellulases and hemicellulases (Puri et al. 2012). This process is based on the development of EAE methods. Nowadays, there are numerous enzyme preparations for different purposes available in the market, which contain various amount of PME, PL, PG, cellulase, and hemicellulase. A successful application of EAE for the extraction of polyphenols from different plant matrices, such as black current juice press residue (Landbo and Meyer 2001), apple skins (Pinelo et al. 2008) and grape skins (Tomaz et al. 2016) is well documented in literature, but there are only a few studies concerning the effect of enzyme addition on the extractability of polyphenols from grape seeds. Chamorro et al. (2012) were investigating the effect of tannase on the content of 0, gallocatechin, epigallocatechin, catechin, epicatechin, procyanidins B1 and B2 as well as on the content of the galloylated forms of epigallocatechin, gallocatechin and epicatechin. They observed that the addition of tannases had a positive effect on the contents of gallic acid, gallocatechin, catechin, epicatechin, procyanidns B1 and B2 and a negative effect on the contents of galloylated forms of epigallocatechin, gallocatechin and epicatechin. This observation could be explained by the tannase’s ability to hydrolyze the ester bonds between flavan-3-ols and gallate. The effect of cellulose, pectinase and tannase on the total seed phenols was studied by Fernandez et al. (2015). They concluded that the addition of these enzymes to the extraction mixture results in an increase of total phenols determined by the Folin–Cioceltau (FC) method. This method is not specific for polyphenols because FC reagent could react with other compounds present in the extracts. The total phenolic content (TPC) determined by the above mentioned method is only a measure of reduction state of the analyzed system. Thus, the obtained values do not necessarily demonstrate the actual state of composition and content of individual polyphenols. In both studies the extractions were performed in conditions described on the enzyme suppliers’ data sheets without optimization.

To the best of our knowledge, the optimization of enzyme-assisted extraction of polyphenols from grape marc seeds using oenological enzyme preparations had never been studied. The literature allows for the assumption that EAE using pectinases and cellulose can be a very efficient non toxic technique for the recovery of phenols from grape seeds. Thus, the objective of this study was the optimization of the extraction conditions for the recovery of polyphenols from grape seeds with the ability to use in industrial application applying different oenological enzyme preparations composed from cellulases and pectinases. For this purpose, Box–Behnken experimental design (BBD) was used with the enzyme dosage, temperature, extraction time and pH as independent experimental variables. The obtained seed extracts were analyzed by the HPLC method to determine the effect of these enzyme preparations on the content of individual grape seed polyphenols. The comparison of the new optimized EAE method was done to evaluate its efficiency.

## Materials and methods

### Chemicals and enzymes

Acetonitrile of HPLC grade was purchased from J. T. Baker (Deventer, The Netherlands). Formic acid, glacial acetic acid, and 85 % orthophosphoric acid were obtained from Fluka (Buchs, Switzerland). Acetone, calcium chloride, boric acid and 1 M sodium hydroxide solution were provided from Kemika (Zagreb, Croatia).

The standards used for the identification and quantification purposes were as follows: epigallocatechin, procyanidin B1 and procyanidin B2 (Extrasynthese, Genay Cedex, France); gallic acid, (−)-epicatechin, (+)-catechin and epicatechin-gallate (Sigma-Aldrich, St. Louis, MO, USA).

Lallzyme HC and Lallzyme EX-V isolated from *Aspergillus niger* were obtained from Lallemand Inc. (Montreal, Canada). The features of these enzyme preparations are given in Table 1.

**Table 1:** Commercial names and main activities of enzyme preparations

Enzyme preparation	Main activities			
	Polygalacturonase (PG U g^−1^)	Pectin liase (PL Ug^−1^)	Pectin methylestherase (PME U g^−1^)	Cellulase and hemicellulase
Lallzyme EX-V	4000	120	1000	+++
Lallzyme HC	3500	100	800	−

### Grape marc seeds preparation

Grape marc samples originating from the vinification of ‘Regent’ were obtained in the year 2014 from the Experimental station Jazbina, Faculty of Agriculture, University of Zagreb, Croatia. Grape seeds were manually separated from skins. The seeds were dried in the oven at 60 °C for 10 h. The dry seeds were ground (Coffee Grinder SMK150, Gorenje, Slovenia) into a fine powder and the powder obtained was stored (2 °C) in a glass container.

### Ultrasound-assisted extraction

The UAE was performed with a slight modification according to the method described by Kallithraka et al. (1995). In brief, grape marc seed powder (125 mg) was extracted with a 10 mL of 70 % aqueous acetone for 5 min in an ultrasonic bath (Sonorex Super RK 100 H, Bandelin Electronic, Berlin, Germany) at the temperature of 50 °C. The extract was centrifuged in a LC-321 centrifuge (Tehtnica, Železnik, Slovenia) for 20 min at 2400×*g* at room temperature. The supernatant was collected, concentrated under a vacuum to remove acetone (40 °C) on a Hei–Vap Advantage G3 rotary evaporator (Heidolph, Schwabach, Germany) and brought to a final volume of 10 mL with eluent A (water/phosphoric acid, 99.5:0.5, v/v). The extract was filtered with Phenex-PTFE 0.20 μm syringe filter (Phenomenex, Torrance, USA) and analyzed by HPLC. All extractions were performed in triplicate.

### Enzyme-assisted extraction

The extraction solvents were composed of the appropriate mass of enzyme preparation dissolved in the buffer of the corresponding pH. The solid-to-solvent ratio was 1:80 g mL^−1^ (125 mg of grape marc seeds powder and 10 mL of the extraction solvent). All extractions were performed in glass vials equipped with PTFE-caps on the magnetic stirrer at 400 rpm. The above-mentioned extraction conditions were constant during the optimization process. Buffers solutions with pH values of 2, 3 and 4 were made based on the method outlined in the literature (Buffer Solutions Other Than Standards 1999). To increase the activity of PME, calcium ions in the final concentration of 0.015 M were added to all buffer solutions. Working solutions of enzyme preparations were prepared daily by dissolution of 50 mg of enzyme preparations in 100 mL of the buffer solution of the corresponding pH. Enzyme dosages were expressed in terms of mg of the enzyme preparation per g of the sample. The enzymes were inactivated by heating (90 °C, 1 min) in a water bath. The extract was centrifuged for 20 min at 2400×*g* at room temperature. The supernatant was collected and brought to a final volume of 10 mL with 0.5 % phosphoric acid.

### Experimental design and statistical analysis

The Box–Behnken experimental design was applied to four independent variables on three levels (Table 2). As responses (*Y*, dependent variables), resulting contents of gallocatechin, procyanidin B1, procyanidin B2, catechin, epicatechin and epigallocatechin were expressed as their sum (flavan-3-ol contents) and content of gallic acid was used. The results of the BBD experiments were studied by non-linear multiple regression with backward elimination to fit the following second-order equation to the dependent *Y* variables:

1 $$Y = B_{0} + \varSigma B_{i} x_{i} + \varSigma B_{ij} x_{i} x_{j} + \varSigma B_{ii} x_{i}^{2} \quad (i = 1, \, 2 \ldots k)$$

*B*_0_, *B*_*i*_, *B*_*ii*_ and *B*_*ij*_ are the parameters for the linear, quadratic and interaction effects, respectively: *x*_*i*_ and *x*_*j*_ are the levels of independent variables in the coded values. The analysis of the experimental design and calculation of the predicted data was done using the Design Expert 9 software (Stat-Ease Inc., Minneapolis, USA). The parameters were interpreted using an *F* test. To establish the optimal conditions for gallic acid content and flavan-3-ols content, analysis of variance (ANOVA), regression analysis and plotting of the response surface plot were conducted.

**Table 2:** Independent factors and their levels used in the response surface design

Factors	Factor levels		
Coded levels	−1	0	1
*X*_1_: enzyme dosage (mg g^−1^)	10	15	20
*X*_2_: pH	2	3	4
*X*_3_: extraction temperature (°C)	40	45	50
*X*_4_: extraction time (h)	1	2	3

The mean values, standard deviations and significant differences of the data were calculated and reported using OriginPro 8 (OriginLab Corporation, Northampton, USA). The results were analyzed using one-way ANOVA and the differences between the means were evaluated by Tukey’s posthoc test at a confidence level of 95 % (*p* < 0.05). The data reported in all of the tables were the average of triplicate observation.

### LC analysis

The separation, identification and quantification of polyphenols from grape marc seeds extracts were performed according to the method described by Tomaz and Maslov (2016) on an Agilent 1100 Series system (Agilent, Germany), equipped with autosampler, column thermostat, diode array detector (DAD), fluorescence detector (FLD) and coupled to an Agilent Chemstation data-processing station. The separation was performed on a reversed-phase column Luna Phenyl-Hexyl [4.6 × 250 mm; 5 μm particle (Phenomenex, Torrance, USA)]. The solvents were water:phosphoric acid (99.5:0.5, v/v, eluent A) and acetonitrile:water:phosphoric acid; 50:49.5:0.5, v/v/v, eluent B). Using DAD, gallic acid was detected at 280 nm. Using FLD, flavan-3-ols were detected at *λ*_ex_ = 225 nm and *λ*_em_ = 320 nm. Quantification of individual polyphenol peaks was completed by using a calibration curve of the corresponding standard compound. Where reference compounds were not available, the calibration of the structurally related compound was used. The results are expressed in mg kg^−1^ of dry weight (d.w.) of grape seeds. For the peak assignment, grape seed extracts were analyzed with an Agilent 1200 Series system (Agilent, Germany) coupled in-line to an Agilent model 6410 mass spectrometer fitted with an ESI source.

## Results and discussion

### Optimization of extraction conditions

Levels of extraction variables were designated based in our previous study (Tomaz et al. 2016) and properties of corresponding pectinases described in the literature. The process parameters and experimental data of 27 runs were presented in Table 3. These runs were separately conducted for individual enzyme preparations, namely Lallzyme HC and Lallzyme EX-V.

**Table 3:** Box–Behnken experimental design (coded)

	Factor 1	Factor 2	Factor 3	Factor 4	Resp. 1 (EX-V)	Resp. 2 (EX-V)	Resp. 1 (HC)	Resp. 2 (HC)
	Enzyme dosage (mg g^−1^)	pH	Temperature (°C)	Time (h)	Flavan-3-ols (mg kg^−1^)	Gallic acid (mg kg^−1^)	Flavan-3-ols (mg kg^−1^)	Gallic acid (mg kg^−1^)
1	15.00	2.00	50.00	2.00	17,860.0	111.87	21,509.6	126.00
2	20.00	3.00	45.00	1.00	18,697.2	162.92	18,260.0	119.12
3	20.00	3.00	50.00	2.00	21,597.7	237.97	21,188.4	152.61
4	10.00	4.00	45.00	2.00	21,719.5	183.65	18,515.4	129.50
5	15.00	4.00	40.00	2.00	19,222.2	185.15	20,070.1	139.20
6	10.00	3.00	45.00	3.00	19,073.1	161.65	18,788.4	139.01
7	20.00	2.00	45.00	2.00	19,826.6	152.67	21,183.4	130.42
8	15.00	3.00	50.00	3.00	18,272.0	217.57	19,612.3	166.09
9	15.00	3.00	45.00	2.00	20,703.3	205.69	20,527.7	147.90
10	10.00	2.00	45.00	2.00	20,664.8	143.16	21,453.9	124.45
11	15.00	3.00	45.00	2.00	19,670.0	190.38	19,965.7	145.11
12	15.00	4.00	45.00	1.00	17,480.0	144.11	20,518.8	126.17
13	15.00	3.00	50.00	1.00	17,675.4	135.10	19,276.1	121.42
14	15.00	4.00	45.00	3.00	20,230.0	156.50	20,172.4	148.88
15	15.00	4.00	50.00	2.00	18,889.5	175.33	19,130.8	140.11
16	15.00	2.00	45.00	3.00	17,257.2	135.92	22,455.1	131.55
17	10.00	3.00	50.00	2.00	19,013.0	180.44	18,138.9	133.33
18	15.00	3.00	40.00	3.00	19,876.7	211.46	21,806.7	159.01
19	20.00	3.00	45.00	3.00	21,127.4	228.58	20,822.3	164.36
20	20.00	4.00	45.00	2.00	19,429.5	175.81	21,165.5	147.99
21	15.00	2.00	45.00	1.00	19,875.4	120.80	21,125.1	117.59
22	15.00	3.00	40.00	1.00	16,212.3	146.52	18,191.0	121.49
23	15.00	3.00	45.00	2.00	20,043.6	199.24	20,073.3	131.63
24	10.00	3.00	45.00	1.00	19,740.0	141.02	20,197.7	114.66
25	20.00	3.00	40.00	2.00	18,107.1	188.13	18,730.0	150.52
26	10.00	3.00	40.00	2.00	21,011.1	189.20	20,916.3	136.73
27	15.00	2.00	40.00	2.00	19,000.5	150.42	20,287.9	120.42

The obtained contents of gallic acid (GA) and flavan-3-ols (FOL), using both enzyme preparations, were best characterized by a quadratic polynomial equation. Parameters for analyzing the variance (ANOVA) of the response variables were depicted in Table 4. Model fine-tuning in terms of the best possible values for *p* values of model and lack of fit, as well, *R* ^2^, adjusted *R* ^2^ and adequacy precision was done by applying backward elimination with alpha out value of 0.5000. This value of alpha out allow retention of some regression coefficients with *p* value higher then 0.05. Regression coefficients of main effects with *p* value higher then 0.05 were required to support hierarchy. Model *p* values for both enzyme preparations were lower than 0.0009 while the lack of fit *p* values was greater than 0.10. These values indicate that the obtained models were accurate. The determination coefficients of 0.87 and 0.83 for FOL and GA, respectively, in a case of preparation EX-V suggested that the model could explain all the variations. The values of these coefficients for both studied groups of phenols in a case of preparation HC were 0.90. Adequacy (Adq) precision measures the signal to noise ratio. A ratio greater than 4 is desirable. For all of the cases examined, the values greater than 9 indicate adequate signals, thus these models can be used to navigate the design space.

**Table 4:** Parameters of analysis of variance (ANOVA) for the fitted model

Response	Flavan-3-ol content		Gallic acid content	
	*p* value	Coefficients	*p* value	Coefficients
EX-V				
Model	0.0008		<0.0001	
Lack of fit	0.3910		0.1775	
*R* ^2^		0.87		0.83
Adj *R* ^2^		0.74		0.75
Adq precision		9.27		12.45
*X*_1_: enzyme dosage	0.3327		0.0195	
*X*_2_: pH	0.3232		0.0022	
*X*_3_: temperature	0.9605		0.8276	
*X*_4_: time	0.0245		0.0003	
*X*_1_ *X*_2_	0.0693			
*X*_1_ *X*_3_	0.0016		0.0426	
*X*_1_ *X*_4_	0.0452		0.1887	
*X*_2_ *X*_3_			0.3945	
*X*_2_ *X*_4_	0.0020			
*X*_3_ *X*_4_	0.0469			
*X*_1_^2^	0.0136			
*X*_2_^2^	0.0794		<0.0001	
*X*_3_^2^	0.0111		0.0035	
*X*_4_^2^	0.0036			
HC				
Model	0.0004		0.0007	
Lack of fit	0.2242		0.7863	
*R* ^2^		0.90		0.90
Adj *R* ^2^		0.79		0.77
Adq precision		11.37		10.91
*X*_1_: enzyme dosage	0.0915		0.0008	
*X*_2_: pH	0.0005		0.0015	
*X*_3_: temperature	0.5425		0.5751	
*X*_4_: time	0.0055		<0.0001	
*X*_1_ *X*_2_	0.0162		0.3138	
*X*_1_ *X*_3_	0.0003			
*X*_1_ *X*_4_	0.0024		0.0187	
*X*_2_ *X*_3_	0.0419			
*X*_2_ *X*_4_	0.1370		0.4870	
*X*_3_ *X*_4_	0.0084			
*X*_1_^2^	0.1998			
*X*_2_^2^	0.0046		0.0040	
*X*_3_^2^	0.1246		0.0411	
*X*_4_^2^			0.3281	

The Table 5 depicts second-order polynomial equations for FOL and GA for both enzyme preparations. The linear effect of time was positive for all dependent variables, which indicates that raising the amount of time had a positive effect on the contents of FOL and GA for both enzyme preparations. This observation could be explained by the structure of grape seeds’ cell walls as well as by the location of polyphenols inside the grape seed cells. A grape seed contains primary and secondary cell walls (Hanlin et al. 2010) thus longer incubation time is mandatory for the achievement of the optimal degradation by pectines as well the diffusion of polyphenolic contents from inside of cells (vacuoles) to the bulk solvent. The polyphenols are located in inner parts of the seed structure so it takes longer to diffuse to the extraction solvent. The temperature increase had a negative effect on the FOL and GA contents for both enzyme preparations (negative linear effect of the temperature). In relation to the enzyme dosage, a positive linear effect was observed for GA (EX-V), FOL (HC) and GA (HC) which shows that the increasing of enzyme dosage improves the extraction of the above mentioned analytes. Lower enzyme dosage had a positive effect on the recovery of FOL in a case of EX-V preparation. Regarding the pH of extraction mixture, only FOL (HC) exhibited negative linear effect.

**Table 5:** Second-order polynomial equations and regression coefficients of the response values

Responses	Second-order polynomial equations
EX-V	
Flavan-3-ols content (mg kg^−1^)	*Y* = 20,139 − 203*x*_1_ + 207*x*_2_ − 10.19*x*_3_ + 513*x*_4_ − 363*x*_1_ *x*_2_ + 1372*x*_1_ *x*_3_ + 774*x*_1_ *x*_4_ +1342*x*_2_ *x*_4_ − 767*x*_3_ *x*_4_ + 687*x*_1_^2^ − 382*x*_2_^2^ − 971*x*_3_^2^ − 1124*x*_4_^2^
Gallic acid content (mg kg^−1^)	*Y* = 198 + 12.25*x*_1_ + 17.14*x*_2_ − 1.05*x*_3_ + 21.77*x*_4_ − 14.65*x*_1_ *x*_3_ + 11.26*x*_1_ *x*_4_ + 7.18*x*_2_ *x*_3_ − 37.84*x*_2_^2^ − 22*x*_4_^2^
HC	
Flavan-3-ols content (mg kg^−1^)	*Y* = 20,189 + 278*x*_1_ − 703*x*_2_ − 95*x*_3_ + 507*x*_4_ + 730*x*_1_ *x*_2_ + 1309*x*_1_ *x*_3_ + 993*x*_1_ *x*_4_ − 540*x*_2_ *x*_3_ − 419*x*_2_ *x*_4_ − 820*x*_3_ *x*_4_ − 321*x*_1_^2^ − 708*x*_2_^2^ − 384*x*_3_^2^
Gallic acid content (mg kg^−1^)	*Y* = 142 + 7.28*x*_1_ + 6.78*x*_2_ − 1.02*x*_3_ + 15.70*x*_4_ + 3.13*x*_1_ *x*_2_ + 5.22*x*_1_ *x*_4_ + 2.19*x*_2_ *x*_4_ − 8.85*x*_2_^2^ + 2.17*x*_3_^2^ − 2.56*x*_4_^2^

### Response surface analysis for the enzyme preparation EX-V

**Fig. 1:**
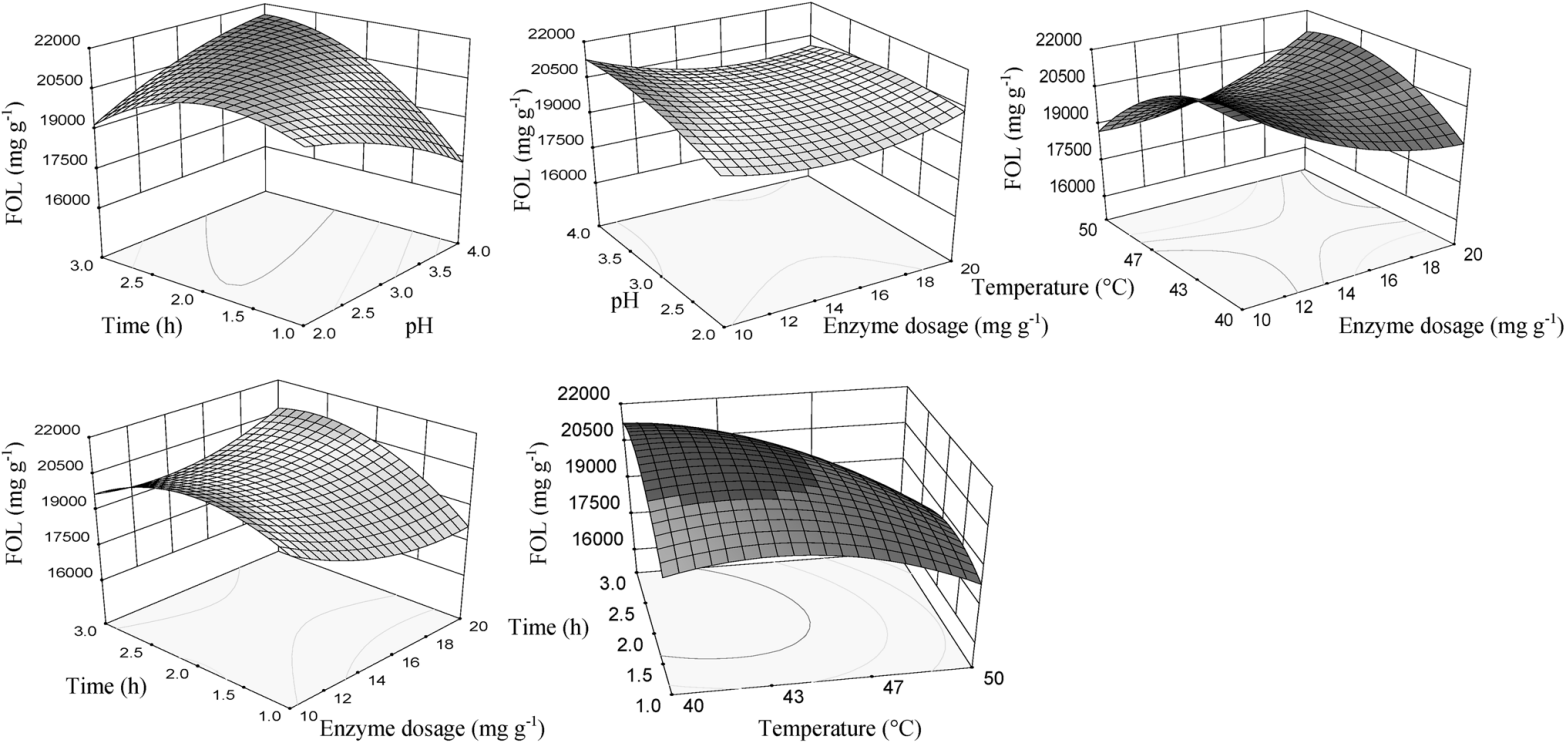
Response surface for flavan-3-ol content (FOL), in function of enzyme dosage, pH, temperature and time of extraction using enzyme preparation Lallzyme EX-V. The value of missing independent variable in each plot was kept at the centre point

The response surfaces for significant effects of the independent variables on FOL content are presented in Fig. 1. Increasing the enzyme dosage with pH and time, as well, results in an increase of flavan-3-ols content. Longer extraction time and lower temperature had a positive effect on the FOL recovery. The interaction effect between the extraction time and pH was positive, indicating that higher pH value together with a longer extraction time results in a better response. Optimum values of the enzyme dosage, pH, the time and temperature for the maximum content of flavan-3-ols were 10–12 mg g^−1^, 3.5–4.0, 2.5–3.0 h and 40–45 °C, respectively.

**Fig. 2:**
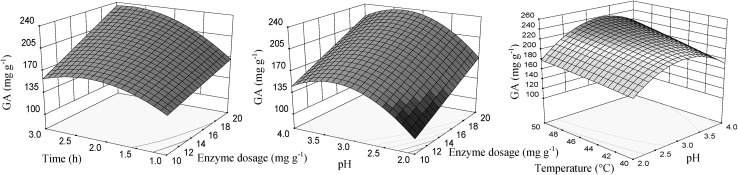
Response surface for gallic acid content (GA), in function of enzyme dosage, pH, temperature and time of extraction using enzyme preparation Lallzyme EX-V. The value of missing independent variable in each plot was kept at the centre point

Significant effects of individual variables on GA content are presented by the response surface plots (Fig. 2). The content of GA was function of pH and the enzyme dosage and the temperature, as well. The interaction effect between extraction time and enzyme dosage was positive. The increase of the extraction time and the enzyme dosage had a positive effect on the extraction of gallic acid. The maximum GA content was achieved with pH in the range between 3 and 3.5, the enzyme dosage of 20 mg g^−1^, the temperature of 50 °C and the time of 3 h.

Based on the obtained results it could be concluded that the optimum extraction conditions using Lallzyme EX-V for recovery of FOL and GA were quite different, especially in terms of the applied temperature and enzyme dosage.

### Response surface analysis for the enzyme preparation HC

**Fig. 3:**
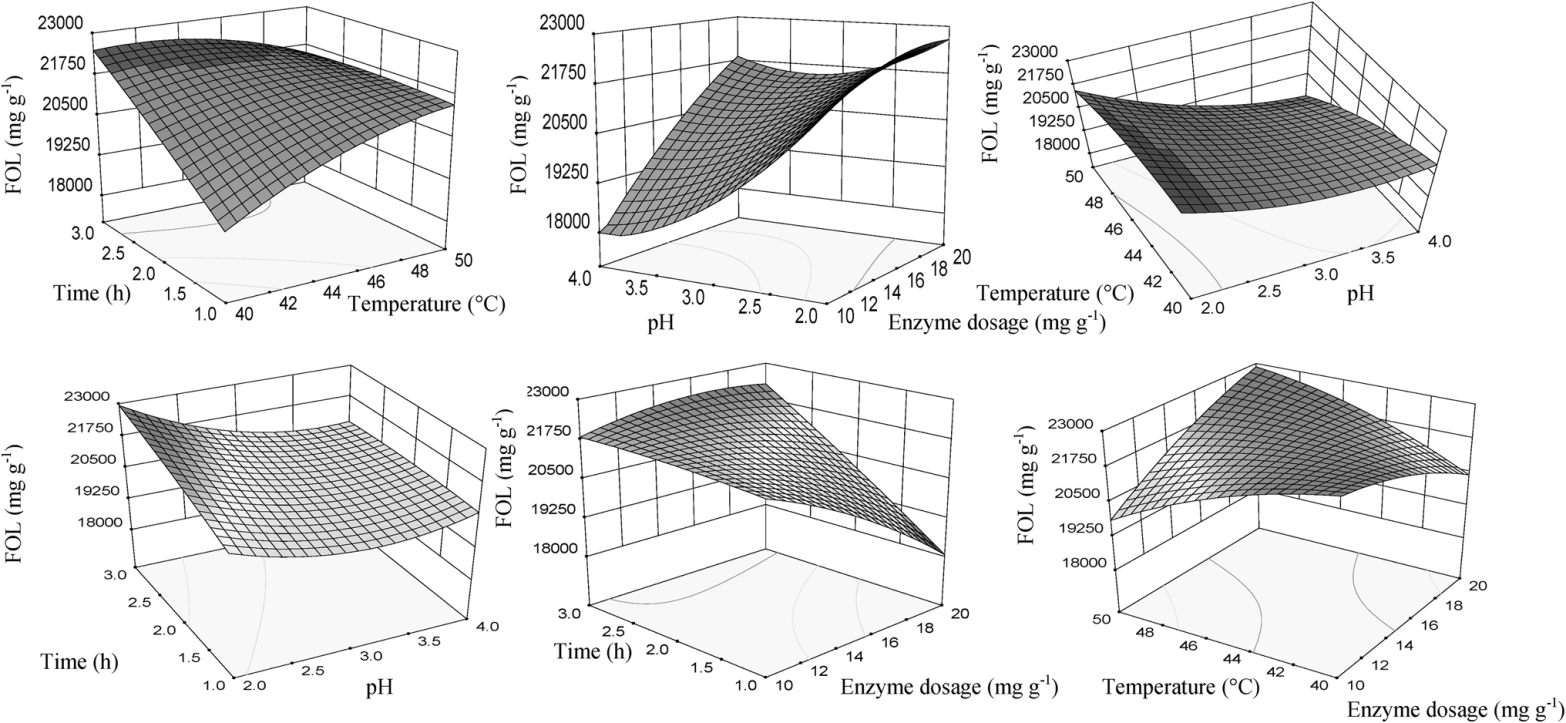
Response surface for flavan-3-ol content (FOL), in function of enzyme dosage, pH, temperature and time of extraction using enzyme preparation Lallzyme HC. The value of missing independent variable in each plot was kept at the centre point

Figure 3 shows the response surfaces plots for the significant effects of the independent variables on FOL content. The interaction between the extraction time and temperature was negative, thus longer extraction time at lower temperature had a positive effect on the FOL content. Increasing the enzyme dosage with the temperature and time, as well, results in an increase of flavan-3-ols content. Flavan-3-ols content was function between pH and the enzyme dosage, temperature and time, as well. Lowering the pH value together with rising temperature, enzyme dosage and extraction time lead to the improvement of flavan-3-ols extraction. The optimum values of the enzyme dosage, pH, time and temperature for obtaining maximum content of flavan-3-ols were 18–20 mg g^−1^, 2.0–2.5, 2.5–3.0 h and 45–50 °C, respectively.

**Fig. 4:**
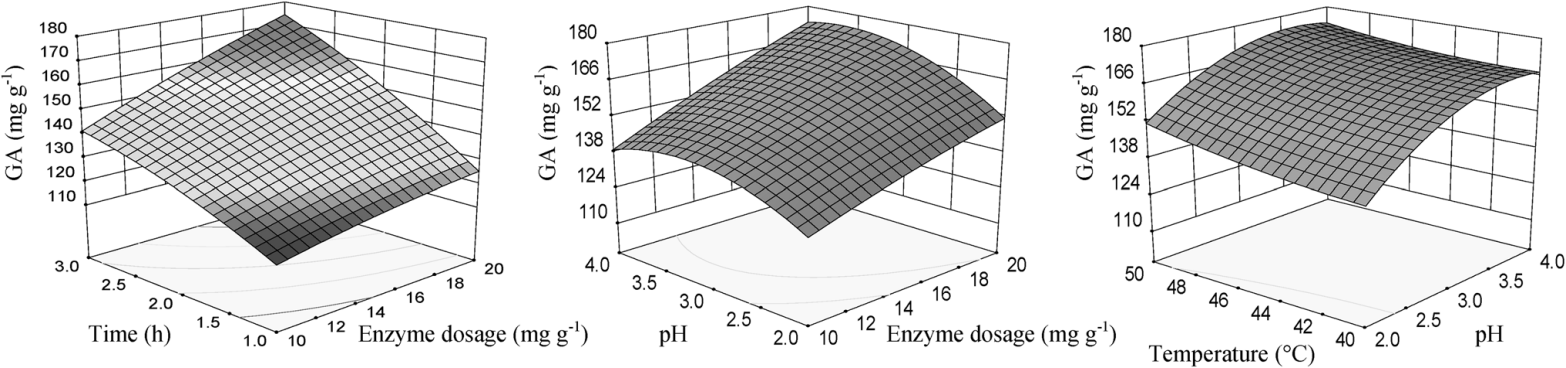
Response surface for gallic acid content (GA), in function of enzyme dosage, pH, temperature and time of extraction using enzyme preparation Lallzyme HC. The value of missing independent variable in each plot was kept at the centre point

Significant effects of the individual variables on GA content are displayed by the response surface plots (Fig. 4). The interaction effects between the enzyme dosage, pH and the extraction time were positive. The increase of the enzyme dosage together with an increase of the time and pH, as well, had a positive effect on the content of GA. The content of gallic acid was function of pH and the temperature. Higher temperature and pH values led to a better recovery of gallic acid. The maximum GA content was achieved with pH in the range between 3.5 and 4, enzyme dosage of 18–20 mg g^−1^, temperature of 40–50 °C and time of 2.5–3 h.

The optimum extraction conditions for recovery of GA and FOL were quite different, especially in terms of pH value.

### Determination and experimental validation of the optimal conditions

**Fig. 5:**
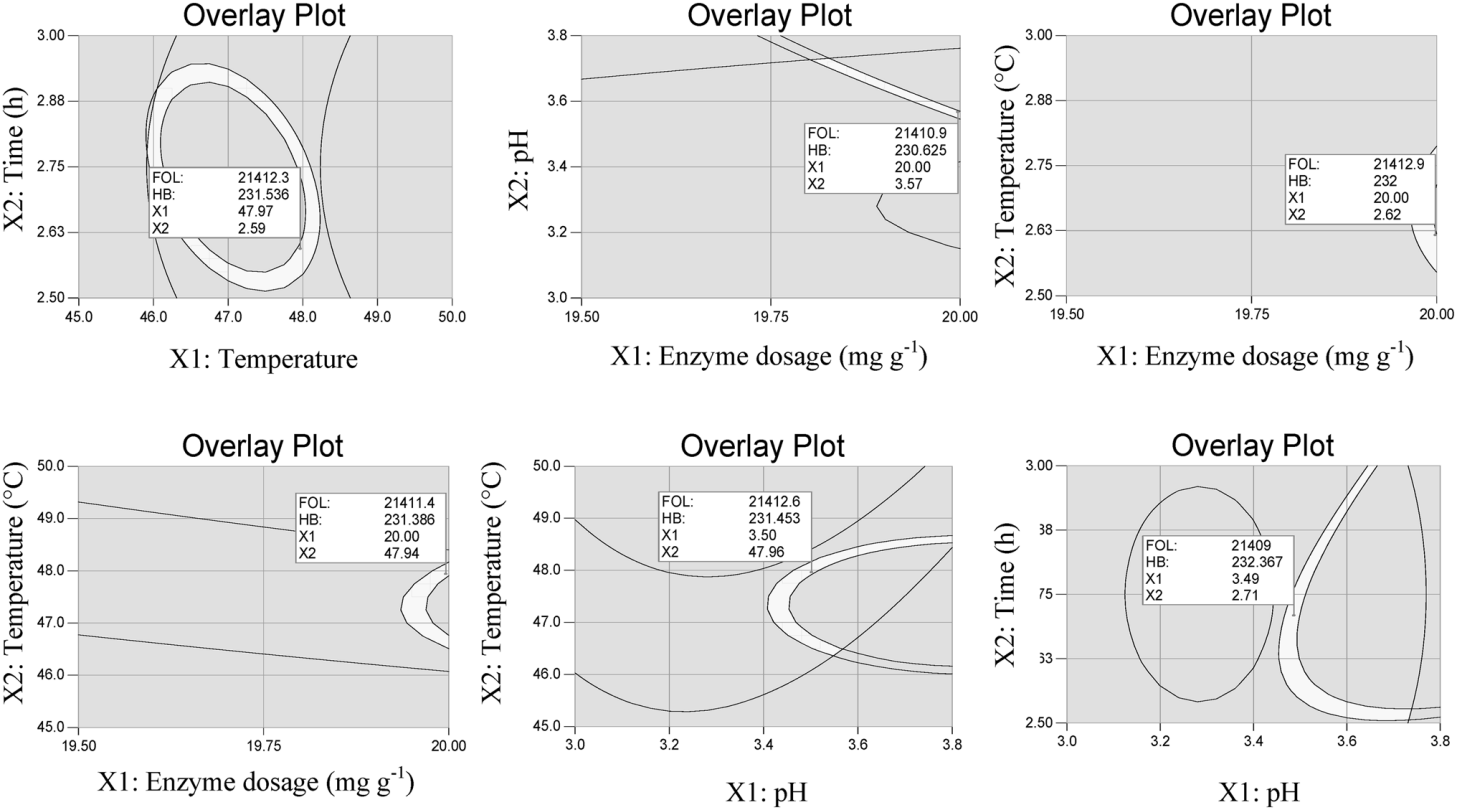
Overlay plot for flavan-3-ol content (FOL) and gallic acid content (GA) in function of enzyme dosage, pH, temperature and time of extraction using enzyme preparation Lallzyme EX-V

**Fig. 6:**
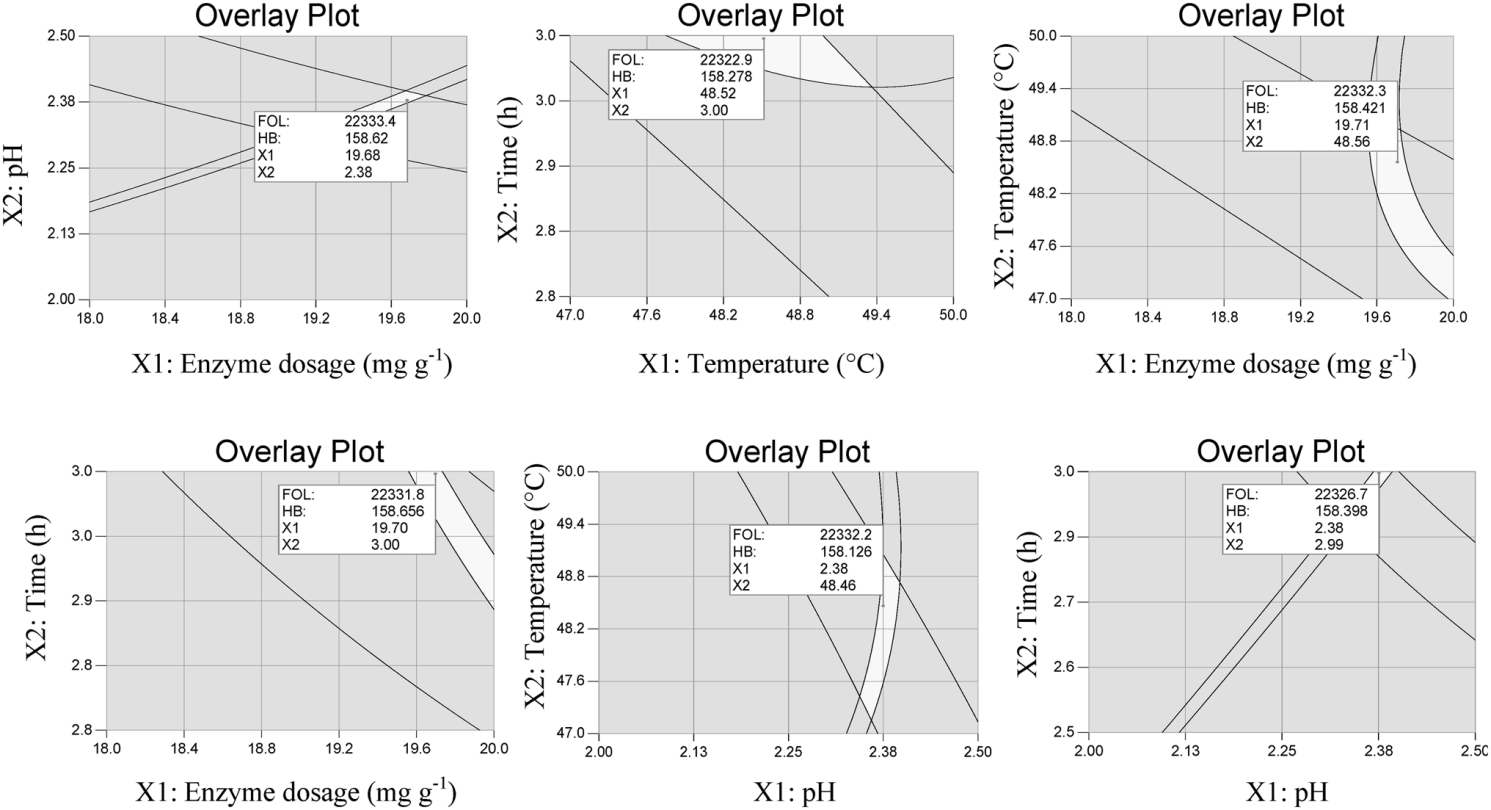
Overlay plot for flavan-3-ol content (FOL) and gallic acid content (GA) in function of enzyme dosage, pH, temperature and time of extraction using enzyme preparation Lallzyme HC

The model’s predictive capacity was proven by the determination of optimal conditions. For this purpose, the simplex method and the maximal possible desirability for the maximum contents of FOL and GA was used. The overlay plots of FOL and GA contents for both enzyme preparations are depicted in Figs. 5 and 6. The unshaded regions on Figs. 5 and 6 represent extraction conditions, where the content of FOL exceeded 21,350 mg kg^−1^ (Fig. 5) and 22,250 mg kg^−1^ (Fig. 6), while the content of GA exceeded 220 mg kg^−1^ (Fig. 5) and 150 mg kg^−1^ (Fig. 6). In regard to Fig. 5 the maximum possible obtained desirability was 0.94. The obtained result can be explained by the different nature of flavan-3-ols and gallic acid which results in obtaining quite different optimum values for individual independent variables. For the enzyme preparation EX-V optimum extraction conditions were as follows: enzyme dosage 20 mg g^−1^, pH 3.55, temperature 48 °C and time 2.60 h. The maximum possible obtained desirability was 0.95 for enzyme preparation HC, while the optimum extraction conditions were as follows: enzyme dosage 20 mg g^−1^, pH 2.38, temperature 48.5 °C and time 3.00 h.

The suitability of model’s equations for the optimum response values is tested using the above mentioned optimal conditions. The experimental values were very close to the predicted ones, consequently indicating that the RSM models were satisfactory and accurate (Table 6).

**Table 6:** Comparison of predicted and observed values

Enzyme preparation	Enzyme dosage (mg g^−1^)	pH	Temperature (°C)	Time (h:min)	Predicted values		Experimental values (*n* = 3)	
					Flavan-3-ols (mg kg^−1^)	Gallic acid (mg kg^−1^)	Flavan-3-ols (mg kg^−1^)	Gallic acid (mg kg^−1^)
EX-V	20.00	3.55	48	2:38	21,413	231.0	21,408 ± 21	227.04 ± 0.35
HC	20.00	2.38	48.5	3:00	22,323	158.4	22,204 ± 41	145.12 ± 0.42

### Comparison of EAE and UAE

The efficiency of the EAE was determined by a comparison of the individual polyphenol contents observed in extracts obtained with extraction solvents, which contained enzyme preparation dosage of 0 and 20 mg g^−1^, while the other extraction conditions were listed in Table 6. The results of this analysis (Table 7) unequivocally confirm that the addition of the enzyme preparation had a significant positive effect on the final content of all analyzed polyphenolic compounds in obtained extracts.

**Table 7:** The content of individual phenols extracted from grape seeds by using different, extraction methods, results are expressed in mg kg^−1^ d.w

Compound	EX-V (controle)^A^		EX-V^B^		HC (controle)^A^		HC^B^		UAE	
	${\tilde{Y}}\pm {\text{SD}}$	RSD	${\tilde{Y}}\pm {\text{SD}}$ ± SD	RSD	${\tilde{Y}}\pm {\text{SD}}$ ± SD	RSD	${\tilde{Y}}\pm {\text{SD}}$ ± SD	RSD	${\tilde{Y}}\pm {\text{SD}}$ ± SD	RSD
Gallicacid	133.40 ± 0.82^a^	0.61	227.04 ± 0.35^b^	0.15	124.01 ± 0.54^c^	0.44	145.12 ± 0.42^d^	0.29	49.73 ± 1.70^e^	3.42
Epigallocatechin	39.46 ± 0.32^a^	0.81	51.39 ± 0.30^b^	0.58	43.36 ± 0.32^c^	0.74	46.41 ± 0.32^d^	0.69	44.47 ± 1.70^c,d^	3.82
Epicatechin- gallate	3.20 ± 0.22^a^	6.88	9.28 ± 0.64^b^	6.90	2.06 ± 0.41^a^	19.90	5.36 ± 0.44^c^	8.40	2.25 ± 1.08^a^	48.00
Gallocatechin	38.74 ± 1.38^a^	3.56	64.38 ± 0.08^b^	0.12	55.43 ± 0.09^c^	0.16	49.18 ± 0.08^d^	0.16	57.51 ± 1.12^c^	1.95
Procyanidin B1	1927.62 ± 3.17^a^	0.16	2646.33 ± 5.36^b^	0.20	22,22.39 ± 7.14^c^	0.32	2279.42 ± 7.33^c^	0.32	2,726.56 ± 110.90^b^	4.07
Catechin	9297.77 ± 20.53^a^	0.22	10,714.18 ± 22.27^b^	0.21	8,856.87 ± 23.51^c^	0.27	12,187.88 ± 32.35^d^	0.27	9074.17 ± 280.54^a,c^	3.09
Procyanidin B4	543.50 ± 7.74^a^	1.42	703.74 ± 3.11^b^	0.44	570.31 ± 1.91^a^	0.33	646.11 ± 2.16^c^	0.33	621.88 ± 30.29^c^	4.87
Procyanidin B2	1739.50 ± 2.84^a^	0.16	2207.00 ± 1.61^b^	0.07	1865.30 ± 3.67^c^	0.20	2177.03 ± 4.28^b^	0.20	2041.41 ± 83.64^d^	4.10
Epicatechin	4082.87 ± 5.87^a^	0.14	5011.35 ± 4.03^b^	0.80	3957.01 ± 5.46^a^	0.14	4812.35 ± 6.65^c^	0.14	4039.48 ± 134.05^a^	3.32
Flavan-3-ol content	17,672.90 ± 24.80^a^	0.14	21,407.65 ± 21.32^b^	0.10	17,572.74 ± 32.63^a^	0.19	22,203.74 ± 41.33^b^	0.19	18,613.79 ± 628.20^d^	3.37

Superscript letters a, b, c, d and e indicate grouping within row. Different letters show statistical difference *p* < 0.05

${\tilde{Y}}\pm$ mean value (*n* = 3), *SD* standard deviation, *RSD* relative standard deviation

^A^Enzyme dosage 0 mg g^−1^; ^B^ enzyme dosage 20 mg g^−1^

The extracts obtained using the preparation EX-V contained the highest amount of nearly all polyphenolic compounds (Table 7). Such observation may be attributed to different portions of each pectinases in various enzyme preparations and the presence of cellulase and hemicellulase in the preparation EX-V. Those enzymes could improve the cleavage of the cell wall, thus enhancing the diffusion of the intracellular contents to the bulk extraction solvent. It is known that a hydrolysis of tannins might occur during extraction in a very acid environment (pH 0–2.5). Grape marc seed tannins are made from the monomeric forms of flavan-3-ols interconnected with flavan bonds. Catechin is the most common terminal unit of grape seed tannins (Mattivi et al. 2009). As there is no steric hindrance, cleavages of the terminal units appear first during tannin hydrolyses. The optimal extraction conditions for the preparation HC are performed in a highly acidic environment (pH 2.40) which could possibly explain a larger content of catechin obtained in that extract. The reproducibility and precision of EAE optimized methods was determined from calculated relative standard deviations. These values for most analyzed compounds were lower than 1 %, thus these methods are precise and reproducible.

To determine the efficiency of the EAE using two enzyme preparations, the Lallzyme EX-V and Lallzyme HC, the content of studied polyphenolic compounds in the obtained extracts was compared with those in the extracts obtained using the ultrasound-assisted extraction. The results strongly suggest that the EAE, regardless of which enzyme preparation was used, is a more efficient method for the extraction of all studied compounds from grape seed than the traditionally used UAE method. By applying the UAE method, the extraction of polymeric forms of flavan-3-ols (tannins) can appear, along with the recovery of simple polyphenols. The chromatograms recorded after the injection of the EAE extracts contained fewer peaks than those obtained after the injection of the UAE extracts. These observations could be related to a greater selectivity of the EAE method. By applying the EAE method, the number of steps during the extraction process was reduced e.g., removing of extraction solvent is not necessary. The thermal inactivation of enzymes does not cause the degradation of polyphenols (data not shown).

The new optimized method does not allow for the recovery of tannins, due to their low polarity and the inability of dissolution in aqueous phases. Ethanol is environmentally friendly and safe for human health, so the grape seed extracts obtained by ethanol could be used in pharmaceutical, food and cosmetic industries. When ethanol was used in the simple solid–liquid extraction, the extraction time was 19 h (Casazza et al. 2010). A shorter extraction time with ethanol as the extraction solvent can be achieved by applying microwave assisted extraction (MAE) or ultrasound assisted extraction (Ghafoor et al. 2009; Li et al. 2011). The application of UAE and MAE requires the acquisition of expensive extraction systems and ethanol as well, whereof the market price is higher than the enzyme preparation one. The preparation of dry phenolic grape seed extracts requires drying. Freeze–drying is one of the most used drying techniques for this purpose. In comparison with ethanolic extracts, longer time is needed for drying aqueous extracts, including EAE extracts.

## Conclusion

The BBD was successfully used to optimize the enzyme-assisted extraction of polyphenols from grape marc seeds. Regardless of the enzyme preparation, the optimized EAE methods are a powerful tool for the extraction of polyphenols from grape marc seeds. Benefits of this technique include the use of environmentally friendly chemicals, and extracts obtained are immediately ready for HPLC analysis as well as for industrial use without the need for the removal of extraction solvents. Grape marc seeds obtained after vinification of the cultivar ‘Regent’ could be used as a commercial source of flavan-3-ols, and especially of catechin and epicatechin.
